# 9-(3-Methoxy­phen­yl)-6,6-dimethyl-4-phenyl-2,3,5,6,7,9-hexa­hydro­thieno[3,2-*b*]quinolin-8(4*H*)-one 1,1-dioxide

**DOI:** 10.1107/S1600536810003600

**Published:** 2010-02-06

**Authors:** Shun-Hua Wang, Yue-Ning Jiang, Jiang-Na Zhang

**Affiliations:** a School of Mechatronic Engineering, Lanzhou Jiaotong University, Lanzhou 730070, People’s Republic of China; b School of Civil Engineering, Lanzhou Jiaotong University, Lanzhou 730070, People’s Republic of China

## Abstract

The title compound, C_26_H_27_NO_4_S, with a  thiophene ring fused to a quinoline ring, was synthesized *via* the condensation of dihydro­thio­phen-3(2*H*)-one 1,1-dioxide, 5,5-dimethyl-3-(phenyl­amino)cyclo­hex-2-enone and 3-methoxy­benzaldehyde in refluxing ethanol. In the crystal structure, the thiophene dioxide ring and the pyridine ring adopt envelope conformations. The connection of the pyridine ring to the phenyl and benzene rings can be described by the C–C–C–C and C–N–C–C torsion angles of 45.5 (2) and 88.7 (2)°, respectively. The cyclo­hex-2-enone ring is in a half-chair conformation. The crystal packing is stabilized by non-classical inter­molecular C—H⋯O hydrogen bonds with the carbonyl O and sulfone O atoms acting as acceptors.

## Related literature

For the use of thienoquinoline compounds as ATP-sensitive potassium channel openers, see: Altenbach *et al.* (2006 ▶); Carroll *et al.* (1999 ▶). For puckering parameters, see: Cremer & Pople (1975 ▶).
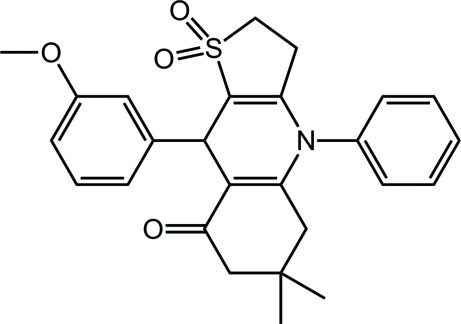


## Experimental

### 

#### Crystal data

C_26_H_27_NO_4_S
*M* *_r_* = 449.55
Monoclinic, 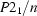

*a* = 11.2488 (14) Å
*b* = 14.8013 (18) Å
*c* = 13.3866 (16) Å
β = 92.747 (7)°
*V* = 2226.3 (5) Å^3^
*Z* = 4
Mo *K*α radiation
μ = 0.18 mm^−1^
*T* = 113 K
0.20 × 0.14 × 0.12 mm

#### Data collection

Rigaku Saturn CCD area-detector diffractometer
Absorption correction: multi-scan (*CrystalClear*; Rigaku/MSC, 2005 ▶) *T*_min_ = 0.965, *T*_max_ = 0.979
14414 measured reflections
4843 independent reflections
3756 reflections with *I* > 2σ(*I*)
*R*_int_ = 0.042

#### Refinement

*R*[*F* ^2^ > 2σ(*F* ^2^)] = 0.049
*wR*(*F* ^2^) = 0.125
*S* = 1.04
4843 reflections
293 parameters
H-atom parameters constrained
Δρ_max_ = 0.24 e Å^−3^
Δρ_min_ = −0.50 e Å^−3^

### 

Data collection: *CrystalClear* (Rigaku/MSC, 2005 ▶); cell refinement: *CrystalClear*; data reduction: *CrystalClear*; program(s) used to solve structure: *SHELXS97* (Sheldrick, 2008 ▶); program(s) used to refine structure: *SHELXL97* (Sheldrick, 2008 ▶); molecular graphics: *SHELXTL* (Sheldrick, 2008 ▶); software used to prepare material for publication: *SHELXTL*.

## Supplementary Material

Crystal structure: contains datablocks I, global. DOI: 10.1107/S1600536810003600/hg2633sup1.cif

Structure factors: contains datablocks I. DOI: 10.1107/S1600536810003600/hg2633Isup2.hkl

Additional supplementary materials:  crystallographic information; 3D view; checkCIF report

## Figures and Tables

**Table 1:** Hydrogen-bond geometry (Å, °)

*D*—H⋯*A*	*D*—H	H⋯*A*	*D*⋯*A*	*D*—H⋯*A*
C22—H22⋯O1^i^	0.95	2.53	3.409 (2)	154
C23—H23⋯O3^ii^	0.95	2.51	3.353 (2)	148

Symmetry codes: (i) 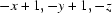; (ii) 
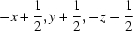
.

**Fig. 1.**
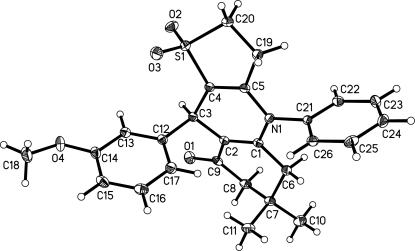
The structure of (I), showing 30% probability displacement ellipsoids and the atom-numbering scheme.

**Fig. 2.**
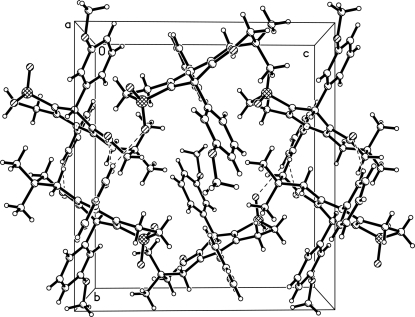
The packing diagram of (I). Intermolecular hydrogen bonds are shown as dashed lines.

## supplementary crystallographic information

### Comment

Thienoquinoline compounds, such as thieno[3,2-*b*]quinoline -1,1-dioxide derivatives, can be uesed as ATP-sensitive Potassium channel opener (Altenbach *et al.*, 2006; Carroll *et al.*, 1999). This led us to pay attention to the synthesis and bioactivity of these compounds. During the synthesis of thieno[3,2-*b*]quinoline derivatives, the title compound, (I) was isolated and its structure was determined by X-ray diffraction. Here we report its crystal structure.

The molecular structure of (I) is shown in Fig. 1. In this structure, the thiophene ring is in envelope conformation, for the deviation of C20 from the C19/C5/C4/S1 plane is 0.386 (3)Å with r.m.s. of 0.000. The pyridine ring adopts a half-boat conformation. Cremer & Pople puckering analysis (Cremer & Pople, 1975) shows that its *Q* is 0.186 (2) Å, θ and φ are 109.4 (6) and 348.5 (6)°, respectively. The connection of the pyridine ring and phenyl rings (C12—C17 and C21–C26) can be described by the C2–C3–C12–C17 and C5–N1–C21–C26 torsion angles of 45.5 (2)° and 88.7 (2)°, respectively. According to Cremer & Pople puckering parameters of the cyclohex-2-enone ring, it is in a half-chair conformation. Its *Q* is 0.490 (2) Å, θ and φ are 121.4 (2)° and 50.0 (3)°, respectively. The crystal packing is stablized by intermolecular nonclassical C—H···O hydrogen bonds with the carbonyl O and sulphone O atoms respectively acting as acceptors.(Fig.2 & Table 1).

### Experimental

The title compound was synthesized by the reaction of dihydrothiophen-3(2*H*)-one-1,1-dioxide (1 mmol), 5,5-dimethyl-3-(phenylamino)cyclohex-2-enone (1 mmol) and 3-methoxybenzaldehyde (1 mmol) in 10 ml ethanol under reluxing until completion (monitored by TLC). Cooling the reaction mixture slowly gave single crystals suitable for X-ray diffraction.

### Refinement

All H atoms were placed in calculated positions, with C—H = 0.95, 0.98, 0.99 or 1.00 Å, and included in the final cycles of refinement using a riding model, with *U*_iso_(H) = 1.2*U*_eq_(parent atom).

### Crystal data

**Table:**

C_26_H_27_NO_4_S	*F*(000) = 952
*M**_r_* = 449.55	*D*_x_ = 1.341 Mg m^−^^3^
Monoclinic, *P*2_1_/*n*	Mo *K*α radiation, λ = 0.71070 Å
Hall symbol: -P 2yn	Cell parameters from 6501 reflections
*a* = 11.2488 (14) Å	θ = 2.1–27.9°
*b* = 14.8013 (18) Å	µ = 0.18 mm^−^^1^
*c* = 13.3866 (16) Å	*T* = 113 K
β = 92.747 (7)°	Prism, colourless
*V* = 2226.3 (5) Å^3^	0.20 × 0.14 × 0.12 mm
*Z* = 4	

### Data collection

**Table:**

Rigaku Saturn CCD area-detector diffractometer	4843 independent reflections
Radiation source: rotating anode	3756 reflections with *I* > 2σ(*I*)
confocal	*R*_int_ = 0.042
Detector resolution: 7.31 pixels mm^-1^	θ_max_ = 27.0°, θ_min_ = 2.1°
ω and φ scans	*h* = −14→14
Absorption correction: multi-scan (*CrystalClear*; Rigaku/MSC, 2005)	*k* = −18→18
*T*_min_ = 0.965, *T*_max_ = 0.979	*l* = −17→17
14414 measured reflections	

### Refinement

**Table:**

Refinement on *F*^2^	Primary atom site location: structure-invariant direct methods
Least-squares matrix: full	Secondary atom site location: difference Fourier map
*R*[*F*^2^ > 2σ(*F*^2^)] = 0.049	Hydrogen site location: inferred from neighbouring sites
*wR*(*F*^2^) = 0.125	H-atom parameters constrained
*S* = 1.04	*w* = 1/[σ^2^(*F*_o_^2^) + (0.0695*P*)^2^] where *P* = (*F*_o_^2^ + 2*F*_c_^2^)/3
4843 reflections	(Δ/σ)_max_ < 0.001
293 parameters	Δρ_max_ = 0.24 e Å^−^^3^
0 restraints	Δρ_min_ = −0.50 e Å^−^^3^

### Special details

**Table:**

Geometry. All e.s.d.'s (except the e.s.d. in the dihedral angle between two l.s. planes) are estimated using the full covariance matrix. The cell e.s.d.'s are taken into account individually in the estimation of e.s.d.'s in distances, angles and torsion angles; correlations between e.s.d.'s in cell parameters are only used when they are defined by crystal symmetry. An approximate (isotropic) treatment of cell e.s.d.'s is used for estimating e.s.d.'s involving l.s. planes.
Refinement. Refinement of *F*^2^ against ALL reflections. The weighted *R*-factor *wR* and goodness of fit *S* are based on *F*^2^, conventional *R*-factors *R* are based on *F*, with *F* set to zero for negative *F*^2^. The threshold expression of *F*^2^ > σ(*F*^2^) is used only for calculating *R*-factors(gt) *etc*. and is not relevant to the choice of reflections for refinement. *R*-factors based on *F*^2^ are statistically about twice as large as those based on *F*, and *R*- factors based on ALL data will be even larger.

### Fractional atomic coordinates and isotropic or equivalent isotropic displacement parameters (Å^2^)

**Table:**

	*x*	*y*	*z*	*U*_iso_*/*U*_eq_	
S1	0.50415 (4)	0.22372 (3)	−0.24228 (3)	0.02432 (15)	
O1	0.67631 (11)	0.38885 (9)	0.10478 (10)	0.0322 (3)	
O2	0.60626 (12)	0.26632 (10)	−0.28274 (10)	0.0340 (4)	
O3	0.51009 (13)	0.12739 (9)	−0.22990 (10)	0.0362 (4)	
O4	0.82561 (12)	0.04112 (9)	0.02547 (11)	0.0367 (4)	
N1	0.29857 (13)	0.34280 (10)	−0.05766 (10)	0.0227 (3)	
C1	0.37176 (16)	0.37342 (12)	0.02191 (12)	0.0204 (4)	
C2	0.49043 (15)	0.35533 (11)	0.02666 (12)	0.0207 (4)	
C3	0.54944 (16)	0.28912 (12)	−0.04180 (12)	0.0203 (4)	
H3	0.6250	0.3166	−0.0640	0.024*	
C4	0.46602 (16)	0.27653 (11)	−0.13172 (12)	0.0203 (4)	
C5	0.35101 (16)	0.29912 (12)	−0.13692 (12)	0.0211 (4)	
C6	0.31187 (16)	0.42871 (13)	0.09903 (12)	0.0261 (4)	
H6A	0.2341	0.4009	0.1120	0.031*	
H6B	0.2965	0.4901	0.0719	0.031*	
C7	0.38522 (16)	0.43672 (13)	0.19812 (12)	0.0253 (4)	
C8	0.51206 (17)	0.46420 (13)	0.17341 (13)	0.0267 (4)	
H8A	0.5100	0.5256	0.1441	0.032*	
H8B	0.5622	0.4666	0.2362	0.032*	
C9	0.56777 (17)	0.40077 (12)	0.10200 (13)	0.0234 (4)	
C10	0.32863 (19)	0.50833 (15)	0.26286 (14)	0.0377 (5)	
H10A	0.3274	0.5664	0.2277	0.057*	
H10B	0.3751	0.5142	0.3263	0.057*	
H10C	0.2470	0.4903	0.2761	0.057*	
C11	0.38867 (18)	0.34685 (14)	0.25481 (14)	0.0322 (5)	
H11A	0.4339	0.3544	0.3187	0.048*	
H11B	0.4270	0.3009	0.2147	0.048*	
H11C	0.3073	0.3278	0.2673	0.048*	
C12	0.57895 (15)	0.19902 (12)	0.00974 (12)	0.0212 (4)	
C13	0.68665 (15)	0.15746 (12)	−0.00304 (13)	0.0223 (4)	
H13	0.7437	0.1860	−0.0425	0.027*	
C14	0.71314 (17)	0.07423 (12)	0.04109 (13)	0.0260 (4)	
C15	0.63009 (18)	0.03180 (13)	0.09773 (14)	0.0301 (5)	
H15	0.6474	−0.0251	0.1278	0.036*	
C16	0.52138 (18)	0.07310 (13)	0.11024 (14)	0.0306 (5)	
H16	0.4640	0.0441	0.1490	0.037*	
C17	0.49544 (17)	0.15579 (13)	0.06716 (14)	0.0272 (4)	
H17	0.4206	0.1835	0.0765	0.033*	
C18	0.8431 (2)	−0.05287 (14)	0.04084 (17)	0.0420 (6)	
H18A	0.8341	−0.0673	0.1115	0.063*	
H18B	0.9233	−0.0696	0.0220	0.063*	
H18C	0.7841	−0.0867	−0.0004	0.063*	
C19	0.27918 (16)	0.27641 (13)	−0.23133 (13)	0.0259 (4)	
H19A	0.2288	0.3283	−0.2531	0.031*	
H19B	0.2272	0.2237	−0.2206	0.031*	
C20	0.36954 (17)	0.25471 (14)	−0.30969 (13)	0.0272 (4)	
H20A	0.3827	0.3082	−0.3522	0.033*	
H20B	0.3406	0.2043	−0.3530	0.033*	
C21	0.17165 (15)	0.35858 (12)	−0.06443 (12)	0.0191 (4)	
C22	0.12586 (16)	0.43661 (12)	−0.10791 (13)	0.0249 (4)	
H22	0.1775	0.4823	−0.1304	0.030*	
C23	0.00324 (17)	0.44714 (14)	−0.11823 (14)	0.0313 (5)	
H23	−0.0294	0.5006	−0.1477	0.038*	
C24	−0.07112 (17)	0.38065 (14)	−0.08602 (14)	0.0310 (5)	
H24	−0.1549	0.3878	−0.0945	0.037*	
C25	−0.02438 (18)	0.30363 (14)	−0.04146 (14)	0.0302 (5)	
H25	−0.0761	0.2582	−0.0186	0.036*	
C26	0.09729 (17)	0.29238 (12)	−0.02991 (13)	0.0243 (4)	
H26	0.1296	0.2397	0.0014	0.029*	

### Atomic displacement parameters (Å^2^)

**Table:**

	*U*^11^	*U*^22^	*U*^33^	*U*^12^	*U*^13^	*U*^23^
S1	0.0256 (3)	0.0268 (3)	0.0206 (2)	0.0037 (2)	0.00181 (18)	−0.00228 (17)
O1	0.0230 (8)	0.0349 (8)	0.0380 (8)	−0.0037 (6)	−0.0065 (6)	−0.0019 (6)
O2	0.0275 (8)	0.0469 (9)	0.0284 (7)	−0.0030 (7)	0.0088 (6)	−0.0039 (6)
O3	0.0512 (10)	0.0241 (8)	0.0331 (7)	0.0076 (7)	−0.0011 (6)	−0.0037 (6)
O4	0.0319 (8)	0.0281 (8)	0.0498 (9)	0.0111 (6)	0.0000 (7)	0.0000 (6)
N1	0.0185 (8)	0.0308 (9)	0.0186 (7)	0.0034 (7)	−0.0010 (6)	−0.0043 (6)
C1	0.0239 (10)	0.0198 (9)	0.0175 (8)	−0.0001 (8)	−0.0004 (7)	0.0021 (7)
C2	0.0239 (10)	0.0200 (9)	0.0180 (8)	−0.0002 (8)	−0.0013 (7)	0.0024 (7)
C3	0.0175 (9)	0.0229 (9)	0.0205 (8)	−0.0008 (8)	0.0004 (7)	0.0013 (7)
C4	0.0229 (10)	0.0201 (9)	0.0179 (8)	0.0007 (8)	0.0009 (7)	0.0011 (7)
C5	0.0233 (10)	0.0233 (10)	0.0166 (8)	0.0001 (8)	−0.0002 (7)	0.0000 (7)
C6	0.0270 (10)	0.0309 (11)	0.0200 (9)	0.0056 (9)	−0.0019 (7)	−0.0029 (7)
C7	0.0289 (10)	0.0285 (10)	0.0184 (8)	−0.0006 (9)	−0.0011 (7)	−0.0010 (7)
C8	0.0335 (11)	0.0257 (10)	0.0205 (8)	−0.0053 (9)	−0.0039 (7)	0.0005 (7)
C9	0.0265 (11)	0.0217 (10)	0.0219 (8)	−0.0033 (8)	−0.0017 (7)	0.0071 (7)
C10	0.0452 (13)	0.0437 (13)	0.0241 (10)	0.0038 (10)	−0.0005 (9)	−0.0104 (9)
C11	0.0328 (11)	0.0409 (12)	0.0229 (9)	−0.0069 (10)	0.0015 (8)	0.0045 (8)
C12	0.0200 (9)	0.0230 (9)	0.0203 (8)	−0.0004 (8)	−0.0030 (7)	−0.0004 (7)
C13	0.0207 (10)	0.0237 (10)	0.0222 (8)	−0.0030 (8)	−0.0016 (7)	0.0000 (7)
C14	0.0281 (10)	0.0224 (10)	0.0266 (9)	0.0040 (9)	−0.0062 (7)	−0.0036 (7)
C15	0.0410 (12)	0.0212 (10)	0.0272 (9)	−0.0015 (9)	−0.0067 (8)	0.0030 (7)
C16	0.0371 (12)	0.0283 (11)	0.0266 (9)	−0.0071 (10)	0.0019 (8)	0.0043 (8)
C17	0.0240 (10)	0.0294 (11)	0.0281 (9)	−0.0008 (9)	0.0007 (8)	0.0015 (8)
C18	0.0487 (14)	0.0300 (12)	0.0463 (13)	0.0140 (11)	−0.0088 (10)	0.0000 (10)
C19	0.0223 (10)	0.0343 (11)	0.0208 (9)	0.0036 (8)	−0.0027 (7)	−0.0048 (7)
C20	0.0294 (11)	0.0332 (11)	0.0190 (8)	0.0032 (9)	−0.0002 (7)	−0.0027 (8)
C21	0.0173 (9)	0.0237 (10)	0.0161 (8)	0.0025 (8)	−0.0004 (6)	−0.0023 (7)
C22	0.0273 (10)	0.0244 (10)	0.0232 (9)	−0.0014 (9)	0.0024 (7)	0.0024 (7)
C23	0.0300 (11)	0.0340 (12)	0.0296 (10)	0.0097 (10)	−0.0028 (8)	0.0008 (8)
C24	0.0183 (10)	0.0429 (12)	0.0319 (10)	0.0014 (9)	0.0007 (8)	−0.0072 (9)
C25	0.0287 (11)	0.0360 (12)	0.0265 (10)	−0.0094 (10)	0.0073 (8)	−0.0042 (8)
C26	0.0284 (11)	0.0238 (10)	0.0209 (8)	0.0010 (8)	0.0018 (7)	0.0000 (7)

### Geometric parameters (Å, °)

**Table:**

S1—O3	1.4367 (14)	C11—H11B	0.9800
S1—O2	1.4388 (14)	C11—H11C	0.9800
S1—C4	1.7453 (17)	C12—C13	1.377 (2)
S1—C20	1.7851 (19)	C12—C17	1.397 (2)
O1—C9	1.232 (2)	C13—C14	1.392 (2)
O4—C14	1.382 (2)	C13—H13	0.9500
O4—C18	1.419 (2)	C14—C15	1.382 (3)
N1—C1	1.391 (2)	C15—C16	1.384 (3)
N1—C5	1.397 (2)	C15—H15	0.9500
N1—C21	1.445 (2)	C16—C17	1.378 (3)
C1—C2	1.360 (2)	C16—H16	0.9500
C1—C6	1.502 (2)	C17—H17	0.9500
C2—C9	1.464 (2)	C18—H18A	0.9800
C2—C3	1.516 (2)	C18—H18B	0.9800
C3—C4	1.502 (2)	C18—H18C	0.9800
C3—C12	1.531 (2)	C19—C20	1.529 (2)
C3—H3	1.0000	C19—H19A	0.9900
C4—C5	1.335 (2)	C19—H19B	0.9900
C5—C19	1.505 (2)	C20—H20A	0.9900
C6—C7	1.533 (2)	C20—H20B	0.9900
C6—H6A	0.9900	C21—C22	1.382 (2)
C6—H6B	0.9900	C21—C26	1.382 (3)
C7—C10	1.527 (3)	C22—C23	1.388 (2)
C7—C11	1.531 (3)	C22—H22	0.9500
C7—C8	1.535 (3)	C23—C24	1.374 (3)
C8—C9	1.499 (2)	C23—H23	0.9500
C8—H8A	0.9900	C24—C25	1.379 (3)
C8—H8B	0.9900	C24—H24	0.9500
C10—H10A	0.9800	C25—C26	1.380 (3)
C10—H10B	0.9800	C25—H25	0.9500
C10—H10C	0.9800	C26—H26	0.9500
C11—H11A	0.9800		
			
O3—S1—O2	116.41 (8)	C7—C11—H11C	109.5
O3—S1—C4	111.02 (8)	H11A—C11—H11C	109.5
O2—S1—C4	110.86 (8)	H11B—C11—H11C	109.5
O3—S1—C20	110.30 (9)	C13—C12—C17	118.86 (17)
O2—S1—C20	111.73 (9)	C13—C12—C3	120.42 (15)
C4—S1—C20	94.40 (8)	C17—C12—C3	120.66 (16)
C14—O4—C18	116.59 (15)	C12—C13—C14	120.95 (16)
C1—N1—C5	118.46 (14)	C12—C13—H13	119.5
C1—N1—C21	122.83 (14)	C14—C13—H13	119.5
C5—N1—C21	118.62 (14)	O4—C14—C15	124.72 (17)
C2—C1—N1	121.02 (15)	O4—C14—C13	115.37 (16)
C2—C1—C6	123.14 (15)	C15—C14—C13	119.89 (17)
N1—C1—C6	115.82 (15)	C14—C15—C16	119.33 (18)
C1—C2—C9	119.43 (16)	C14—C15—H15	120.3
C1—C2—C3	123.79 (15)	C16—C15—H15	120.3
C9—C2—C3	116.78 (15)	C17—C16—C15	120.80 (18)
C4—C3—C2	106.85 (14)	C17—C16—H16	119.6
C4—C3—C12	111.52 (14)	C15—C16—H16	119.6
C2—C3—C12	112.49 (14)	C16—C17—C12	120.16 (18)
C4—C3—H3	108.6	C16—C17—H17	119.9
C2—C3—H3	108.6	C12—C17—H17	119.9
C12—C3—H3	108.6	O4—C18—H18A	109.5
C5—C4—C3	125.27 (15)	O4—C18—H18B	109.5
C5—C4—S1	110.17 (13)	H18A—C18—H18B	109.5
C3—C4—S1	124.45 (13)	O4—C18—H18C	109.5
C4—C5—N1	121.31 (16)	H18A—C18—H18C	109.5
C4—C5—C19	117.90 (15)	H18B—C18—H18C	109.5
N1—C5—C19	120.79 (15)	C5—C19—C20	105.97 (15)
C1—C6—C7	113.35 (15)	C5—C19—H19A	110.5
C1—C6—H6A	108.9	C20—C19—H19A	110.5
C7—C6—H6A	108.9	C5—C19—H19B	110.5
C1—C6—H6B	108.9	C20—C19—H19B	110.5
C7—C6—H6B	108.9	H19A—C19—H19B	108.7
H6A—C6—H6B	107.7	C19—C20—S1	106.42 (12)
C10—C7—C11	108.85 (15)	C19—C20—H20A	110.4
C10—C7—C6	108.84 (15)	S1—C20—H20A	110.4
C11—C7—C6	111.20 (15)	C19—C20—H20B	110.4
C10—C7—C8	110.82 (16)	S1—C20—H20B	110.4
C11—C7—C8	109.59 (15)	H20A—C20—H20B	108.6
C6—C7—C8	107.54 (14)	C22—C21—C26	120.93 (17)
C9—C8—C7	113.13 (15)	C22—C21—N1	120.58 (16)
C9—C8—H8A	109.0	C26—C21—N1	118.45 (16)
C7—C8—H8A	109.0	C21—C22—C23	118.96 (17)
C9—C8—H8B	109.0	C21—C22—H22	120.5
C7—C8—H8B	109.0	C23—C22—H22	120.5
H8A—C8—H8B	107.8	C24—C23—C22	120.34 (18)
O1—C9—C2	120.68 (16)	C24—C23—H23	119.8
O1—C9—C8	120.96 (16)	C22—C23—H23	119.8
C2—C9—C8	118.34 (16)	C23—C24—C25	120.17 (18)
C7—C10—H10A	109.5	C23—C24—H24	119.9
C7—C10—H10B	109.5	C25—C24—H24	119.9
H10A—C10—H10B	109.5	C24—C25—C26	120.24 (18)
C7—C10—H10C	109.5	C24—C25—H25	119.9
H10A—C10—H10C	109.5	C26—C25—H25	119.9
H10B—C10—H10C	109.5	C25—C26—C21	119.34 (17)
C7—C11—H11A	109.5	C25—C26—H26	120.3
C7—C11—H11B	109.5	C21—C26—H26	120.3
H11A—C11—H11B	109.5		
			
C5—N1—C1—C2	−5.1 (2)	C3—C2—C9—O1	4.0 (2)
C21—N1—C1—C2	178.44 (16)	C1—C2—C9—C8	1.6 (2)
C5—N1—C1—C6	173.45 (15)	C3—C2—C9—C8	−177.86 (14)
C21—N1—C1—C6	−3.0 (2)	C7—C8—C9—O1	−150.05 (16)
N1—C1—C2—C9	169.93 (15)	C7—C8—C9—C2	31.8 (2)
C6—C1—C2—C9	−8.5 (3)	C4—C3—C12—C13	102.89 (19)
N1—C1—C2—C3	−10.6 (3)	C2—C3—C12—C13	−137.09 (17)
C6—C1—C2—C3	170.97 (16)	C4—C3—C12—C17	−74.5 (2)
C1—C2—C3—C4	19.8 (2)	C2—C3—C12—C17	45.5 (2)
C9—C2—C3—C4	−160.73 (14)	C17—C12—C13—C14	−0.7 (3)
C1—C2—C3—C12	−102.88 (19)	C3—C12—C13—C14	−178.12 (16)
C9—C2—C3—C12	76.58 (19)	C18—O4—C14—C15	20.7 (3)
C2—C3—C4—C5	−16.6 (2)	C18—O4—C14—C13	−160.75 (17)
C12—C3—C4—C5	106.73 (19)	C12—C13—C14—O4	−177.86 (16)
C2—C3—C4—S1	167.50 (12)	C12—C13—C14—C15	0.7 (3)
C12—C3—C4—S1	−69.21 (19)	O4—C14—C15—C16	178.14 (17)
O3—S1—C4—C5	−101.22 (14)	C13—C14—C15—C16	−0.3 (3)
O2—S1—C4—C5	127.76 (13)	C14—C15—C16—C17	−0.1 (3)
C20—S1—C4—C5	12.50 (14)	C15—C16—C17—C12	0.2 (3)
O3—S1—C4—C3	75.25 (16)	C13—C12—C17—C16	0.2 (3)
O2—S1—C4—C3	−55.77 (17)	C3—C12—C17—C16	177.65 (17)
C20—S1—C4—C3	−171.03 (15)	C4—C5—C19—C20	−15.2 (2)
C3—C4—C5—N1	3.8 (3)	N1—C5—C19—C20	164.53 (16)
S1—C4—C5—N1	−179.73 (14)	C5—C19—C20—S1	22.30 (18)
C3—C4—C5—C19	−176.43 (16)	O3—S1—C20—C19	93.82 (14)
S1—C4—C5—C19	0.0 (2)	O2—S1—C20—C19	−135.03 (13)
C1—N1—C5—C4	8.5 (2)	C4—S1—C20—C19	−20.51 (14)
C21—N1—C5—C4	−174.82 (16)	C1—N1—C21—C22	87.6 (2)
C1—N1—C5—C19	−171.20 (16)	C5—N1—C21—C22	−88.9 (2)
C21—N1—C5—C19	5.4 (2)	C1—N1—C21—C26	−94.8 (2)
C2—C1—C6—C7	−18.6 (2)	C5—N1—C21—C26	88.66 (19)
N1—C1—C6—C7	162.88 (15)	C26—C21—C22—C23	−1.1 (3)
C1—C6—C7—C10	169.02 (16)	N1—C21—C22—C23	176.38 (15)
C1—C6—C7—C11	−71.1 (2)	C21—C22—C23—C24	−0.3 (3)
C1—C6—C7—C8	48.9 (2)	C22—C23—C24—C25	1.2 (3)
C10—C7—C8—C9	−174.50 (15)	C23—C24—C25—C26	−0.7 (3)
C11—C7—C8—C9	65.35 (19)	C24—C25—C26—C21	−0.7 (3)
C6—C7—C8—C9	−55.65 (19)	C22—C21—C26—C25	1.6 (3)
C1—C2—C9—O1	−176.51 (16)	N1—C21—C26—C25	−175.93 (15)

### Hydrogen-bond geometry (Å, °)

**Table:**

*D*—H···*A*	*D*—H	H···*A*	*D*···*A*	*D*—H···*A*
C22—H22···O1^i^	0.95	2.53	3.409 (2)	154
C23—H23···O3^ii^	0.95	2.51	3.353 (2)	148

Symmetry codes: (i) −*x*+1, −*y*+1, −*z*; (ii) −*x*+1/2, *y*+1/2, −*z*−1/2.
